# Development of purification process for dual‐function recombinant human heavy‐chain ferritin by the investigation of genetic modification impact on conformation

**DOI:** 10.1002/elsc.202000105

**Published:** 2021-06-19

**Authors:** Shuang Yin, Bingyang Zhang, Jianying Lin, Yongdong Liu, Zhiguo Su, Jingxiu Bi

**Affiliations:** ^1^ School of Chemical Engineering & Advanced Materials Faculty of Engineering, Computer and Mathematical Sciences University of Adelaide Adelaide Australia; ^2^ College of Biomedical Engineering Taiyuan University of Technology Taiyuan P. R. China; ^3^ State Key Laboratory of Biochemistry Engineering Institute of Process Engineering Chinese Academy of Sciences Beijing P. R. China

**Keywords:** ferritin E‐helix turnover, ferritin genetic modification, human heavy‐chain ferritin, protein characterization, protein purification

## Abstract

Ferritin is a promising drug delivery platform and has been functionalized through genetic modifications. This work has designed and expressed a dual‐functional engineered human heavy‐chain ferritin (HFn) with the inserted functional peptide PAS and RGDK to extend half‐life and improve tumor targeted drug delivery. A facile and cost‐effective two‐step purification pathway for recombinant HFn was developed. The genetic modification was found to affect HFn conformation, and therefore varied the purification performance. Heat‐acid precipitation followed by butyl fast flow hydrophobic interaction chromatography (HIC) has been developed to purify HFn and modified HFns. Nucleic acid removal reached above 99.8% for HFn and modified HFns. However, HFn purity reached above 95% and recovery yield (overall) above 90%, compared with modified HFns purity above 82% and recovery yield (overall) above 58%. It is interesting to find that the inserted functional peptides significantly changed the molecule conformation, where a putative turnover of the E‐helix with the inserted functional peptides formed a “flop” conformation, in contrast with the “flip” conformation of HFn. It could be the cause of fragile stability of modified HFns, and therefore less tolerant to heat and acid condition, observed by the lower recovery yield in heat‐acid precipitation.

**Abbreviations**
CD: circular dichroism
CV: column volume
DLS: dynamic light scattering
FT: flow through
HCPs: host cell proteins
HFn: human heavy‐chain ferritin
HIC: hydrophobic interaction chromatography
IEC: ion‐exchange chromatography
IF: Intrinsic fluorescence
PB: phosphate buffer
PBS: phosphate buffered saline
PI: isoelectric point
SEC: size‐exclusion chromatography
TEM: transmission electron microscopy

## 1 INTRODUCTION

Ferritin, a 12 nm nanocage composed of 24 subunits, has been widely adopted as a drug delivery platform as its unique structure, desirable stability and inexpensive assembly expression in *Escherichia coli* (*E. coli*) [1]. In order to improve its performance, researchers have engineered ferritin through peptide fusion with a wide range of functions, including specific affinity for targeted delivery, florescence labeling, and extended half‐life in circulation [2, 3].

**PRACTICAL APPLICATION**

In this study, a cost‐effective two‐step purification pathway was developed for human heavy‐chain ferritin (HFn). Two genetically modified HFns were compared with HFn in purification performance. Structure comparison of HFn and modified HFns was conducted to analyze how the modification impact conformation and purification performance. Results show that PAS peptide insertion probably caused an E helix turnover, and a significant stability decrease was observed in heat‐acid precipitation. Butyl hydrophobic interaction chromatography (HIC) was found to be significantly more effective compared with Q ion exchange chromatography (IEC) in nucleic acid removal. PAS peptide also enlarged HFn hydrodynamic radius. RGDK in HFn‐PAS‐RGDK had a negligible impact on HFn‐PAS conformation, but improved in vitro performance of doxorubicin by targeting tumor cells.

This work can benefit researchers who are interested in developing robust purification process to achieve high‐quality recombinant HFn by fully understanding the mechanism behind molecule conformation change and therefore adopt the process, so‐called product related process.

In ferritin production from *E. coli*, the removal of host cell proteins (HCPs) and nucleic acid are crucial to preparation of high‐quality self‐assembled ferritin for in vivo and pre‐clinical application. Conventional purification process for recombinant ferritin is facing two challenges: 1) typical process is neither time‐efficient nor cost‐effective, and 2) genetic modifications can cause ferritin conformation change and lead to adjustments.

The high thermal stability of ferritin has inspired researchers to heat *E. coli* lysate to precipitate HCPs. Also its nanometer‐scale size allows for a usage of size‐exclusion chromatography (SEC) and ultra‐centrifugation to separate HCPs. After the removal of HCPs, ion exchange chromatography (IEC), and DNase and RNase treatment are commonly used to remove host cell nucleic acid. Li et al. used a 70°C 15 min heating of *E. coli* lysate followed by sucrose density ultra‐centrifugation or ammonium sulfate (AS) precipitation and SEC to purify recombinant HFn and light‐chain ferritin [4, 5]. Masuda et al. did the heating of lysate at 60°C for 10 min and then AS precipitation, prior to IEC and SEC for HFn purification [6]. A four‐step purification pathway comprising 70°C lysate heating, Hitrap Q high performance IEC, ultra‐centrifugation and AS precipitation was adopted in two previous studies [7, 8]. In other research, lysate heating followed by AS precipitation, plus DNase and RNase treatment and SEC were performed to remove HCPs and nucleic acid [9, 10, 11]. Currently, most ferritin purification pathways comprise three to four steps in order to reach desirable purity for in vitro and in vivo tests. Typical recovery yield was 80–100 mg ferritin per 1 L culture [10, 12]. Time‐consuming steps such as ultracentrifugation and SEC, and cost‐inefficient step as DNase and RNase treatment are frequently included.

The purification process of modified ferritins was generally directly adopted from ferritin [12, 13]. However, the molecule conformation change led by modifications and the conformation change impacts on purification performance were not clearly investigated. For example, IEC replaced lysate heating at 60°C, 15 min for purification of GE11 peptide fused ferritin [14]. This is probably due to a stability decrease after fusion. AS concentration in precipitation decreased to 30‐50% from 40‐60% saturation in mutated HFn purification [15]. It is likely to result from the hydrophobicity difference of the mutant. Overall, the adjustments in purification pathway are specific to the modifications. Therefore, a sound understanding of the impact on ferritin of peptide insertion would benefit the development of a robust purification process.

In this study, two modified HFns with C‐terminal fusion of functional peptide, PAS and PAS‐RGDK, were expressed by *E. coli*, named as HFn‐PAS and HFn‐PAS‐RGDK, respectively. PAS peptide is composed of three types of residues, P, A, and S. It was designed by Schlapschy, aiming to mimic poly ethylene glycol (PEG) [16]. It is highly hydrophilic and has a hydration ability to increase molecule hydrodynamic volume to extend half‐life in circulation. In a previous study, Kim has fused 40‐ and 75‐residue PAS peptide to HFn N‐terminal and increased half‐life in circulation by 4.6 and 5.8 times [17]. In this study, 40‐residue PAS peptide was adopted. Another functional peptide RGDK is a tetrapeptide. It belongs to Tumor Penetration Peptide (TPP) and possesses dual‐function as it enhances drug tumor delivery and drug distribution inside whole tumor tissue instead of only tumor cells alongside tumor vessels [18]. Expression form was analyzed by transmission electron microscopy (TEM).

A two‐step, cost‐effective purification pathway has been established to remove HCPs and nucleic acid from HFn, HFn‐PAS and HFn‐PAS‐RGDK. pH and temperature conditions in the first purification step, heat‐acid precipitation, were screened and compared between HFn and modified HFns. In second step, HIC and IEC were compared in terms of efficiency of nucleic acid removal. Furthermore, the impacts of the insertion of functional peptides on HFn conformation were investigated by circular dichroism (CD), intrinsic fluorescence spectroscopy (IF), dynamic light scattering (DLS) and 5% native‐PAGE. In vitro tests were conducted to prove the successful equipment of engineered HFn with the desirable function of tumor targeting.

## 2 MATERIALS AND METHODS

### 2.1 Materials

Three genes encoding HFn, HFn‐PAS, and HFn‐PAS‐RGDK, respectively, were synthesized by the BGI Company (China). HFn‐PAS was constructed by fusing GFLG ASPAAPAPASPAAPAPSAPAASPAAPAPASPAAPAPSAPA GGSGG to HFn subunit C‐terminus through a flexible 15‐residue linker (GGGSGGGTGGGSGGG). Forty underlined residues constitute PAS peptide and GFLG is a Cathepsin B cleavable site. HFn‐PAS‐RGDK has four extra C‐terminus residues, RGDK, compared with HFn‐PAS. Three types of genes were inserted between NdeІ and XhoІ site of pET30a plasmid, and plasmids were then transformed into *Escherichia coli (E. coli)* BL21 (DE3) using heat‐shock transformation method.

Tryptone, yeast extract, Isopropyl β‐d‐thiogalactoside (IPTG) and kanamycin were purchased from Thermo Scientific (USA). Chromatography columns were bought from GE healthcare (USA). Doxorubicin hydrochloride (DOX) was purchased from Dalian Meilun Biotechnology (China). MCF7 cell line was kindly offered by Dr Nicholas Eyre from the University of Adelaide. RPMI‐1640 medium, penicillin‐streptomycin solution (100×), fetal bovine serum (FBS), and 0.25% trypsin‐EDTA (1×) solution were purchased from Thermo scientific (USA). All other reagents of analytical grade were purchased from Chem‐supply (Australia). Milli Q water was obtained from a Millipore purification system (Merck, USA) and used in all buffers and solutions.

### 2.2 Protein expression and TEM analysis

*E. coli* strains bearing plasmids expressing HFn, HFn‐PAS, and HFn‐PAS‐RGDK were first seeded in conical flasks containing Luria‐Bertani (LB) medium and 100 µg/mL kanamycin and cultured at 37°C for 14 h with shaking at 200 rpm. The cells in the exponential phase were transferred to fresh LB medium supplemented with 100 µg/mL kanamycin and continued to grow in the same condition for 4 h. IPTG was injected into the medium with a final concentration of 0.5 mM for induction. After 4 h, cells were harvested by centrifugation at 4000 rpm, 4°C for 20 min. Harvested cell pellets were re‐suspended using 20 mM phosphate buffer (PB), 2 mM EDTA, pH 7 and subjected to ultra‐sonication for cell disruption. The supernatant of cell lysate following centrifugation (10,000 rpm, 30 min, 4°C) were kept for purification.

To check if all three HFn‐based proteins were expressed as self‐assemblies, TEM analysis was adopted. Five microliter of the supernatant of bacterial cell lysate (0.5 mg/mL) was applied to carbon‐coated, glow‐discharged EM grids and negatively stained with 2% uranyl acetate. Micrographs were recorded on a TVIPS F224HD 2k × 2k CCD camera (Gauting, Germany) using a FEI Tecnai G2 Spirit TEM (Eindhoven, The Netherlands), operating at 100 kV.

### 2.3 Removal of HCPs by heat‐acid precipitation

A two‐step purification, heat‐acid precipitation followed by chromatography, was compared between HFn and modified HFns.

A screening of the buffer pH and heating temperature in heat‐acid precipitation of HFn, HFn‐PAS, and HFn‐PAS‐RGDK was conducted to find the optimal conditions to obtain high protein purity and recovery yield. Briefly, 0.5 mL bacterial lysate (supernatant) (8.0 mg/mL) was mixed with 200 mM acetic acid‐sodium acetate, 2 M NaCl, pH 4.0, 4.5, and 5.0 buffer with a volume ratio of 1:1 and then the mixture was heated at 50°C or 60°C for 5 min. Following the heating, the mixture was centrifuged at 12,000 rpm, 4°C for 15 min to remove precipitates. Supernatant protein concentrations were measured through Bradford assay (Bio‐Rad, USA). Twelve percent reducing SDS‐PAGE (Bio‐Rad, USA) was used to analyze the protein compositions in supernatants. Densitometry scan was used to determine the purity of target protein after purification. Software Image J was adopted [19]. Recovery yield (step) was calculated using Equation 1. In heat‐acid precipitation, “the amount of target protein at purification stage n” means the amount of target protein in heat‐acid precipitation supernatant. “The amount of target protein at purification stage n‐1” means the target protein amount in sample before heat‐acid precipitation.

(1) $$\text{Recovery}\;\text{yield}\left(\text{step}\right)\left(\%\right)=100\%\times \frac{\mathrm{the}\;\mathrm{amount}\;\mathrm{of}\;\mathrm{target}\;\mathrm{protein}\;\mathrm{at}\;\mathrm{purification}\;\mathrm{stage}\;n}{\mathrm{the}\;\mathrm{amount}\;\mathrm{of}\;\mathrm{target}\;\mathrm{protein}\;\mathrm{at}\;\mathrm{purification}\;\mathrm{stage}\;n-1}$$

### 2.4 Nucleic acid removal by chromatography

Anion IEC and HIC were compared in this step to remove nucleic acids and polish target protein purity. AKTA pure (GE healthcare, USA) was employed for all following chromatography.

#### 2.4.1 Nucleic acid removal by IEC

In IEC, Hitrap Q fast flow (FF) column (GE Healthcare, USA), was employed for HFn, HFn‐PAS and HFn‐PAS‐RGDK. pH 7, 8 and 9 were examined. Equilibration buffers and elution buffers at pH 7 and 8 were 20 mM PB without and with 1 M NaCl. 20 mM Tris‐HCl without and with 1 M NaCl buffers were pH 9 equilibration and elution buffer. Supernatants obtained after heat‐acid precipitation underwent Hitrap G25 desalting chromatography (GE Healthcare, USA) into corresponding equilibration buffer before being loaded onto Q FF column. Protein loading amount in each chromatography run was 10 mg. After sample loading and flow through (FT) peak, the column was eluted from 0 to 1 M NaCl linearly within five column volume (CV). Absorbance at 280 and 260 nm were recorded. Flow rate was 1 mL/min. Collected peaks underwent Bradford assay, nucleic acid concentration determination by Quan‐iT 1× dsDNA HS assay kit (Invitrogen, Thermo Fisher Scientific, USA) and 12% reducing SDS‐PAGE analysis. Purity was obtained from densitometry scan and recovery yield (step) was calculated as in Equation 1. In chromatography step, “the amount of target protein at purification stage n” means the amount of target protein in peak. “The amount of target protein at purification stage n − 1” means the target protein amount in loading sample. Nucleic acid removal was calculated as in Equation 2.

(2) $$\begin{array}{c} \text{Nucleic}\;\text{acid}\;\text{removal}\;\left(\%\right)=100\;\left(\%\right)\times \frac{\mathrm{loading}\;\mathrm{sample}\;\mathrm{nucleic}\;\mathrm{acid}\;\mathrm{amount}\;(\mathrm{mg})-\mathrm{final}\;\mathrm{product}\;\mathrm{nucleic}\;\mathrm{acid}\;\mathrm{amount}\;(\mathrm{mg})}{\mathrm{loading}\;\mathrm{sample}\;\mathrm{nucleic}\;\mathrm{acid}\;\mathrm{amount}\;(\mathrm{mg})} \end{array}$$

(3) $$\mathrm{Recovery}\;\mathrm{yield}\;\left(\mathrm{overall}\right)\;\left(\%\right)=100\%\times \frac{\mathrm{the}\;\mathrm{amount}\;\mathrm{of}\;\mathrm{target}\;\mathrm{protein}\;\mathrm{after}\;\mathrm{chromatography}}{\mathrm{the}\;\mathrm{amount}\;\mathrm{of}\;\mathrm{target}\;\mathrm{protein}\;\mathrm{in}\;\mathrm{bacterial}\;\mathrm{lysate}\;(\mathrm{supernatant})}$$

##### Nucleic acid removal by HIC

HIC screening was conducted for HFn and HFn‐PAS‐RGDK. The optimal condition was applied to the HFn‐PAS. Prior to HIC, the supernatants after heat‐acid precipitation were diluted five times with 100 mM PB, 1.2 M AS, incubated at 4°C for 0.5 h and then centrifuged at 12,000 rpm, 4°C, 15 min. During HIC, absorbance at 260 and 280 nm was recorded. Hitrap Octyl FF or Hitrap Butyl FF column (GE Healthcare, USA) was equilibrated with 100 mM PB, 1.0 M AS, pH 6.5 before sample loading. After FT peak finished, column was eluted with 0 to 100% elution buffer (20 mM PB, pH 6.5), one CV gradient. Flow rate was 1 mL/min. HIC peaks were collected and protein concentrations were determined using Bradford assay. Protein purities in peaks were analyzed using densitometry scan of SDS‐PAGE gel. Recovery yield (step) and nucleic acid removal were calculated using Equations 1 and 2. Recovery yield (overall), the recovery yield after heat‐acid precipitation and chromatography, was calculated as in Equation 3.

#### 2.4.2 Structural comparison of purified HFn and modified HFns

##### Secondary and tertiary structural comparison

Purified HFn‐based proteins were buffer exchanged into 20 mM PB, pH 7 and their concentrations were adjusted to 0.2 mg/mL for CD and IF analysis. CD spectroscopy was measured on a J‐810 spectrometer (Jasco, Japan) at 25°C using a 1.0 mm path length quartz cuvette. The wavelength scanning was conducted from 260 to 190 nm at a rate of 500 nm/min with a bandwidth of 1 nm. The average of three scans of each sample was presented. IF spectroscopy was performed on F‐4500 fluorescence spectrophotometer (Hitachi, Japan). The excitation wavelength was 280 nm and the emission was recorded from 300 to 400 nm with a scanning rate of 1200 nm/min. 1.0 cm path length cuvette was used. Each sample was also subjected to scan for three times.

##### Nanoparticle structural comparison

Purified HFn, HFn‐PAS and HFn‐PAS‐RGDK were buffer exchanged into 20 mM PB, pH 7 through Hitrap G25 desalting column (GE Healthcare, USA). Protein hydrodynamic sizes and zeta potentials were measured on a Zetasizer Nano ZS90 (Malvern, UK). Before measurement, purified protein samples were adjusted to 0.5 mg/mL and centrifuged at 10,000 rpm, 4°C for 20 min. Instrument was equilibrated at 25°C and every sample was measured three times. Six micrograms of each protein was loaded to 5% Native‐PAGE gel and run at 90 V in Bio‐Rad electrophoresis set (USA). Loading buffer and gel pH were 6.8.

#### 2.4.3 Drug loading and cell tests

A thermally‐induced passive drug loading approach was applied for all HFn‐based proteins. In brief, 1 mg/mL HFn, HFn‐PAS or HFn‐PAS‐RGDK in 20 mM PB, pH 7 was mixed with 0.2 mg/mL DOX and heated at 50°C for 6 h to load DOX onto HFn‐based proteins. Following that, the heated mixture was centrifuged at 12,000 rpm, 10 min to remove precipitates. The supernatants underwent buffer exchange into phosphate‐buffered saline (PBS) using Hitrap G25 desalting column and unloaded DOX was removed simultaneously in this process. DOX loaded protein peaks were collected for cell tests. Drug loading ratio was calculated as in Equation 4.

(4) $$\begin{array}{ccc} \mathrm{Drug}\;\mathrm{loading}\;\mathrm{ratio} & = & \frac{\mathrm{number}\;\mathrm{of}\;\mathrm{DOX}}{\mathrm{number}\;\mathrm{of}\;\mathrm{nanocage}}\\ & = & \frac{C_{\mathrm{DOX}}•Mw_{\mathrm{nanocage}}}{C_{\mathrm{nanocage}}•Mw_{\mathrm{DOX}}} \end{array}$$

*C* is concentration (mg/mL), and *M*_w_ is molecular weight. The DOX concentration (*C*_DOX_) and the concentration of protein nanocage (*C*_nanocage_) were determined by measuring absorbance at 480 and 280 nm, respectively. Because DOX has absorbance at both 280 and 480 nm but protein only has absorbance at 280 nm, we assume OD480_DOX/nanocage_ = OD480_DOX_; OD280_DOX/nanocage_ = OD280_DOX_ + OD280_nanocage_. Standard OD vs. C linear curves of DOX and HFn‐based proteins were determined by serial concentrations of DOX (1‐40 µg/mL) and protein nanocages (0.1 − 1.2 mg/mL).

In cell tests, MCF7, a human breast cancer cell line was used. MCF7 cells were cultured in RPMI‐1640 medium with 1% penicillin‐streptomycin and 10% fetal bovine serum at 37°C in an atmosphere of 95% air and 5% carbon dioxide. Intracellular distribution assay aimed to investigate if loaded DOX could be released from HFn‐based proteins to function after entering tumor cells. 1 × 10^5^ MCF7 cells in exponential phase were seeded in each well of 12‐well plates and incubated for 24 h for attachment. Free DOX or DOX loaded HFn, HFn‐PAS, HFn‐PAS‐RGDK (10 µg/mL DOX equivalent) in serum free RPMI‐1640 medium were incubated with cells for 3 h. Cell control well was incubated with serum‐free RPMI‐1640 medium. Drugs were then removed and wells were washed with PBS before the addition of complete medium for another 36 h incubation. Subsequently, cells in wells were fixed with 4% formaldehyde and washed with PBS for three times. Hoechst 33258 was added to wells to stain cell nucleus and then washed away with PBS for another three times. Cells were observed under Bio‐Rad ZOE fluorescence cell imager (USA). Blue channel (Excitation: 355/40 nm, Emission: 433/36 nm) was used for locating cell nucleus and green channel (Excitation: 480/17 nm, Emission: 517/23 nm) was used for observing drug distribution.

Cytotoxicity assay was performed to compare IC_50_ of each DOX loaded HFn‐based proteins. Exponentially growing MCF7 cells were seeded in 96‐well plates at a density of 1 × 10^4^ cells/well and incubated for 24 h. After that, free DOX and DOX loaded HFn, HFn‐PAS and HFn‐PAS‐RGDK samples with different DOX concentrations (30, 6, 1.2, 0.24, 0.048, 0.0096 µg/mL) were incubated with cells for 60 h, prior to MTT assay to detect the cell viabilities in wells. Each sample was repeated in three wells. Three wells that had not been incubated with drugs and three cell‐free wells with complete medium were applied as 100% viability and 0% viability controls, respectively. Absorbance at 595 and 630 nm of all wells were recorded using Biotek microplate reader (USA) to calculate the IC_50_ of each sample. Absorbance and cell viability of each well were calculated by the following equations.

(5) $$A_{\mathrm{well}}=A_{595}-A_{630}$$

(6) $$\begin{array}{ccc} \mathrm{Cell}\;\mathrm{viability}\;\left(\%\right) & = & \left(A_{\mathrm{well}}-A_{\mathrm{blank}}\right)/\\ & & \left(A_{\mathrm{cell}}-A_{\mathrm{blank}}\right)\times 100\left(\%\right) \end{array}$$

### 2.5 Statistical analysis

Data in cytotoxicity assay are presented as mean ± standard deviation. The statistical significance was assessed via unpaired T test. IC_50_ values were calculated using Origin 9.0 software.

## 3 RESULTS AND DISCUSSION

### 3.1 HFn, HFn‐PAS, and HFn‐PAS‐RGDK expression by *E. coli*

**FIGURE 1:**
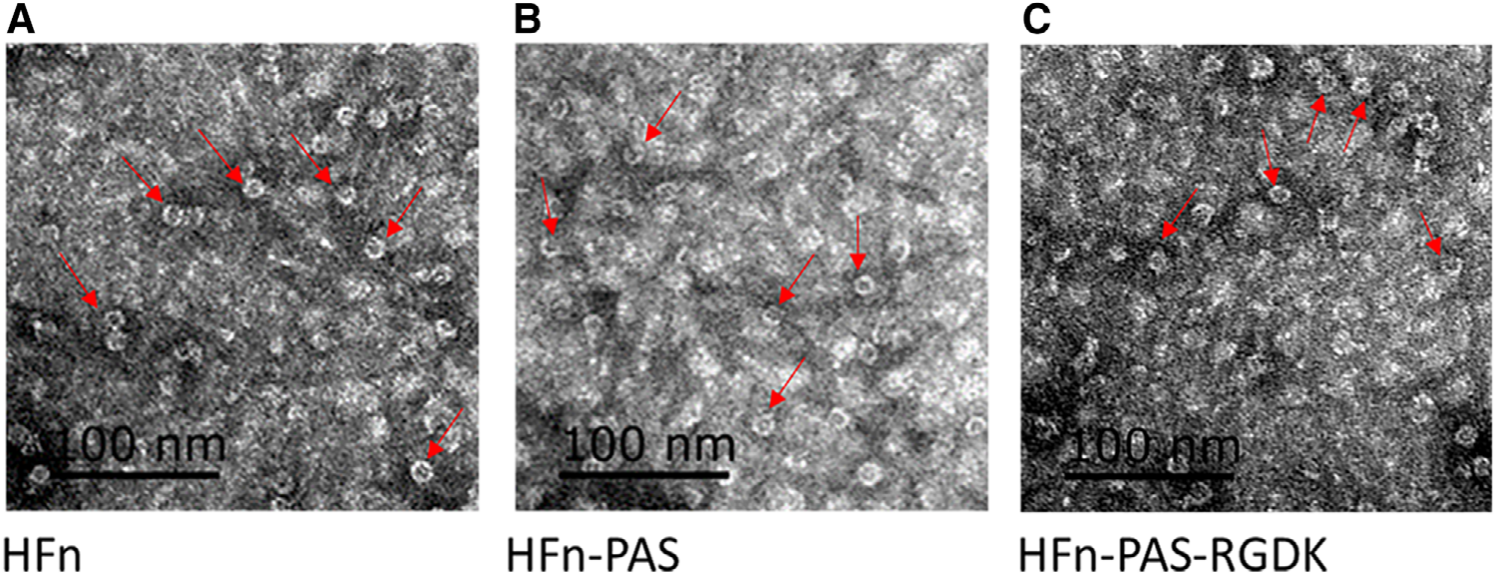
TEM images of bacterial lysates (supernatants) containing HFn or modified HFns. (A) HFn. (B) HFn‐PAS. (C) HFn‐PAS‐RGDK. Red arrows indicate some of the spheres

After 2 L shake flask fermentation, approximate 8 g of wet *E. coli* cells were harvested for each bacterial strain. Supernatant from cell lysate was loaded to SDS‐PAGE to analysis the soluble protein expression level (Figure S1). In Figure S1, SDS‐PAGE result shows that HFn, HFn‐PAS and HFn‐PAS‐RGDK were expressed in both soluble and insoluble forms in *E. coli*, according to their theoretical molecular weights (Mws) (HFn subunit: 21 kDa, HFn‐PAS subunit: 26.0 kDa, HFn‐PAS‐RGDK subunit: 26.5 kDa). All proteins have high soluble expression levels, HFn (33.81% of total protein), HFn‐PAS (32.18% of total protein) and HFn‐PAS‐RGDK (29.86% of total protein), calculated in software Image J. In Figure 1A‐C, hollow spherical structures were observed in all three types of bacterial lysates (supernatants) to indicate the success of self‐assembly. The diameters of hollow spheres were all close to 12 nm, the theoretical diameter of HFn. This indicates neither of the inserted functional peptides affected the folding and assembly of HFn.

### 3.2 Optimization of HCPs removal by heat‐acid precipitation

**FIGURE 2:**
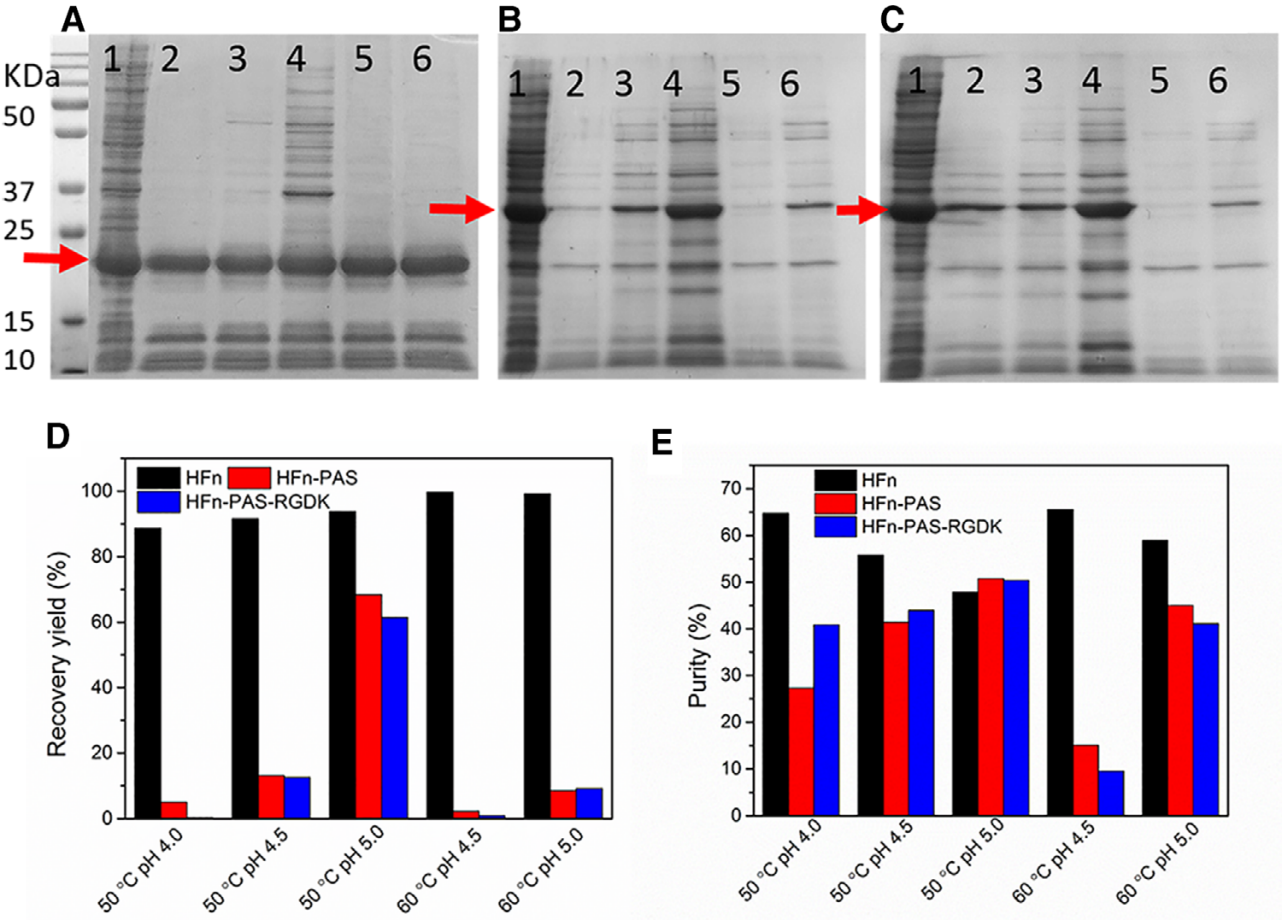
Heat‐acid precipitation optimization of HFn and modified HFns. (A) SDS‐PAGE image for HFn heat‐acid precipitation. (B) SDS‐PAGE image for HFn‐PAS heat‐acid precipitation. (C) SDS‐PAGE image for HFn‐PAS‐RGDK heat‐acid precipitation. Red arrows indicate the target protein bands. Lane 1: bacterial lysate (supernatant); lane 2: 50°C pH 4.0 heat‐acid precipitation supernatant; lane 3: 50°C pH 4.5 heat‐acid precipitation supernatant; lane 4: 50°C pH 5.0 heat‐acid precipitation supernatant; lane 5: 60°C pH 4.5 heat‐acid precipitation supernatant; lane 6: 60°C pH 5.0 heat‐acid precipitation supernatant. (D) Recovery yield (step) of HFn and modified HFns in heat‐acid precipitation. (E) Purity of HFn and modified HFns in heat‐acid precipitation

After cell disruption, supernatants of bacterial cell lysate underwent heat‐acid precipitation to remove HCPs from HFn and modified HFns. SDS‐PAGE images after heat‐acid precipitation are shown in Figure 2A‐C). Recovery yield (step) and purity are in Figure 2D,E. Detailed data are shown in Table S1. Compared with bacterial lysate (supernatant), the majority of HFn was retained in supernatant under all tested conditions. HFn shows the decreasing purity as pH increases at both temperatures (Figure 2E). This is because HFn has a higher thermal and pH stability than most of HCPs, it remained in the supernatant after thermal and acidic treatment while HCPs precipitated (Figure 2A). As the decrease of pH, the amount of precipitated HCPs increased and thus the purity of HFn increased. HFn achieved the best purities under 50°C pH 4.0 (64.77%) and 60°C pH 4.5 (65.50%) (Figure 2A,E). Because of the higher recovery yield (step) in 60°C pH 4.5 (99.69%) than in 50°C pH 4.0 (88.70%) (Figure 2A,D), 60°C pH 4.5 was chosen for the first step purification of HFn.

Interestingly, two modified HFn show the increasing purity with the increase in pH (Figure 2E). The reason is that compared with HFn, two modified HFns have a significantly weaker resistance against the combination of acidic pH and heat. At the conditions of pH 4.0, 50°C and pH 4.5, 60°C, modified HFns cannot resist the acid condition and precipitate together with HCPs. No obvious target protein can be observed in supernatant indicated by Figure 2B,C lane 2 and 5. The purities therefore, were very low. As pH increased to the level which modified HFns could resist, the modified HFns remained in supernatant, whilst the majority of HCPs precipitated and the purity of modified HFns increased. Recovery yield (step) of modified HFns was in the range of 1% to 15% under all tested conditions, except 50°C pH 5.0 (above 60%), in contrast with that of HFn, above 85% at all tested conditions (Figure 2D). Particularly, at 60°C pH 4.5, recovery yield (step) of HFn was around 100%.

Another interesting observation is that the heat‐acid tolerance of HFn‐PAS‐RGDK with two inserted peptides was generally weaker than that of 1 peptide inserted HFn‐PAS. This significant stability drop resulting from insertion of foreign peptides has been reported several times in different proteins [20, 21]. However, how the inserted peptide impacted the stability still requires further investigation.

Based on the recovery yield (step) and purity in Figure 2D,E, the purity of modified HFns were 50.77% (HFn‐PAS) and 50.41% (HFn‐PAS‐RGDK), and the recovery yield (step) was around 75%. Five minutes heating at pH 5.0 and 50°C was selected as the first step condition for two modified HFns. Compared with the optimal condition of HFn (60°C, pH 4.5), the temperature and pH condition for modified HFns (pH 5.0, 50°C) is milder to achieve around 25% lower recovery yield (step) and 15% lower purity.

### 3.3 Removal of nucleic acid by chromatography

#### 3.3.1 Nucleic acid removal by ion‐exchange chromatography (IEC)

After heat‐acid precipitation, host cell nucleic acid could be digested into small fragments, but remained in supernatants with target proteins. Therefore, the second step focused on the nucleic acid removal.

Ion exchanger has been commonly applied to separate protein and nucleic acids based on the differences of their binding strengths [22]. In this work, an anion exchanger, Q FF, was selected, because of the protein stability on acid condition and the isoelectric point (pI) of HFn and modified HFns. HFn nanocage remains stable in pH range of 3.4‐10 [23]. Modified HFns have a significant lower tolerance against acidic pH, as is observed in heat‐acid precipitation. HFn pI is around 4.5‐5.0 [24]. HFn‐PAS and HFn‐PAS‐RGDK predicted pI values are 5.2 and 5.2, calculated by Thermo Fisher Scientific peptide synthesis and proteotypic peptide analyzing tool. If using cation exchanger, the buffer pH in use should be below 4.5 and modified HFns nanocage may not be able to withstand. As a result, Q FF and pH 7, 8 and 9 were selected to separate nucleic acid and HFn based on binding strength difference.

**FIGURE 3:**
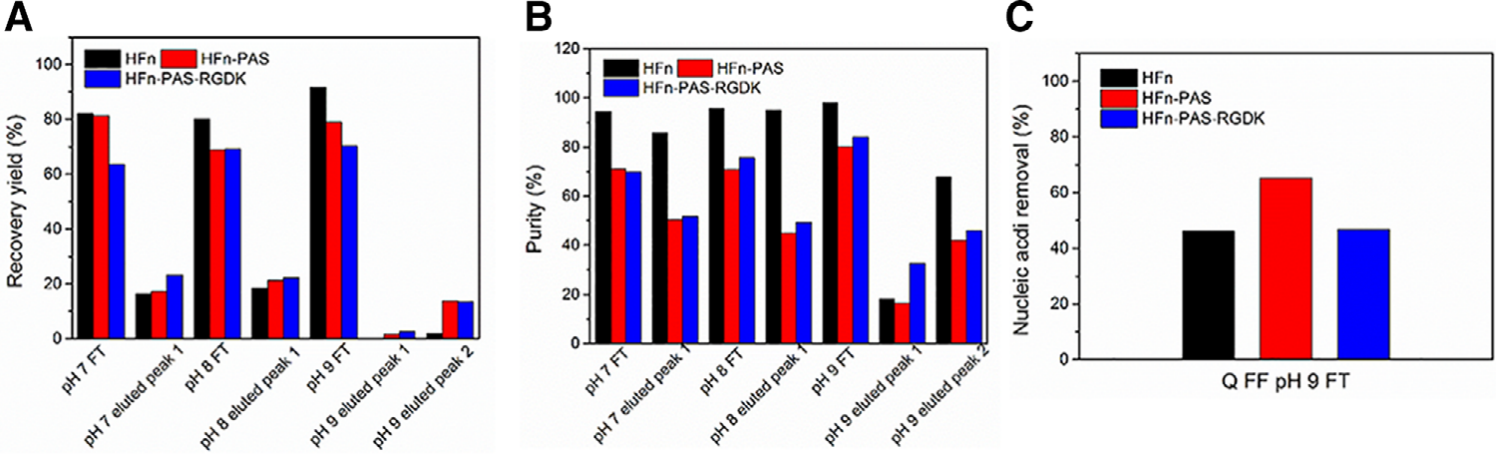
Q FF chromatography purification results of HFn and modified HFns. (A) Recovery yields (step) of FT peaks and elutes peaks from pH 7, 8, and 9 Q FF chromatography. (B) Purity of FT peaks and elutes peaks from pH 7, 8, and 9 Q FF chromatography. (C) Nucleic acid removal percentages of pH 9 FT peaks

Theoretically, HFn and two modified HFns can bind to Q FF because of their acidic pI. As is shown in Figure 3A, surprisingly, in all tested pH values, the majority (65‐92%) of three proteins were in FT peaks, especially HFn. This shows a very weak binding of HFn and modified HFns to Q FF column. All FT and elution peaks had higher OD260 than OD280 values, which indicates the presence of nucleic acid in both FT and elution peaks (Figure S2). The nucleic acid removal was not satisfactory. In pH 9 FT peaks, nucleic acid removal was 46.00% of HFn, 65.17% of HFn‐PAS and 46.81% of HFn‐PAS‐RGDK (Figure 3C). Figure S3 is the SDS‐PAGE images of peaks from Q FF IEC. Table S2 details the purity, recovery yield (step) and nucleic acid removal data.

The observation that all three HFns appeared in FT peaks in tested pH shows that nano‐meter sized proteins are hard to diffuse inside non‐porous resin spheres to interact with the functional groups on resin and the binding is generally weaker compared with small sized proteins, because of the lower value of surface net charge/weight [25, 26]. What is more, HFn is more negatively charged in inner surface than outer surface because more acidic residues were buried inside HFn assembly [27]. Porous IEC resins theoretically can be a feasible means to separate nucleic acid from target proteins than non‐porous IEC resin.

#### 3.3.2 Nucleic acid removal by hydrophobic interaction chromatography (HIC)

**FIGURE 4:**
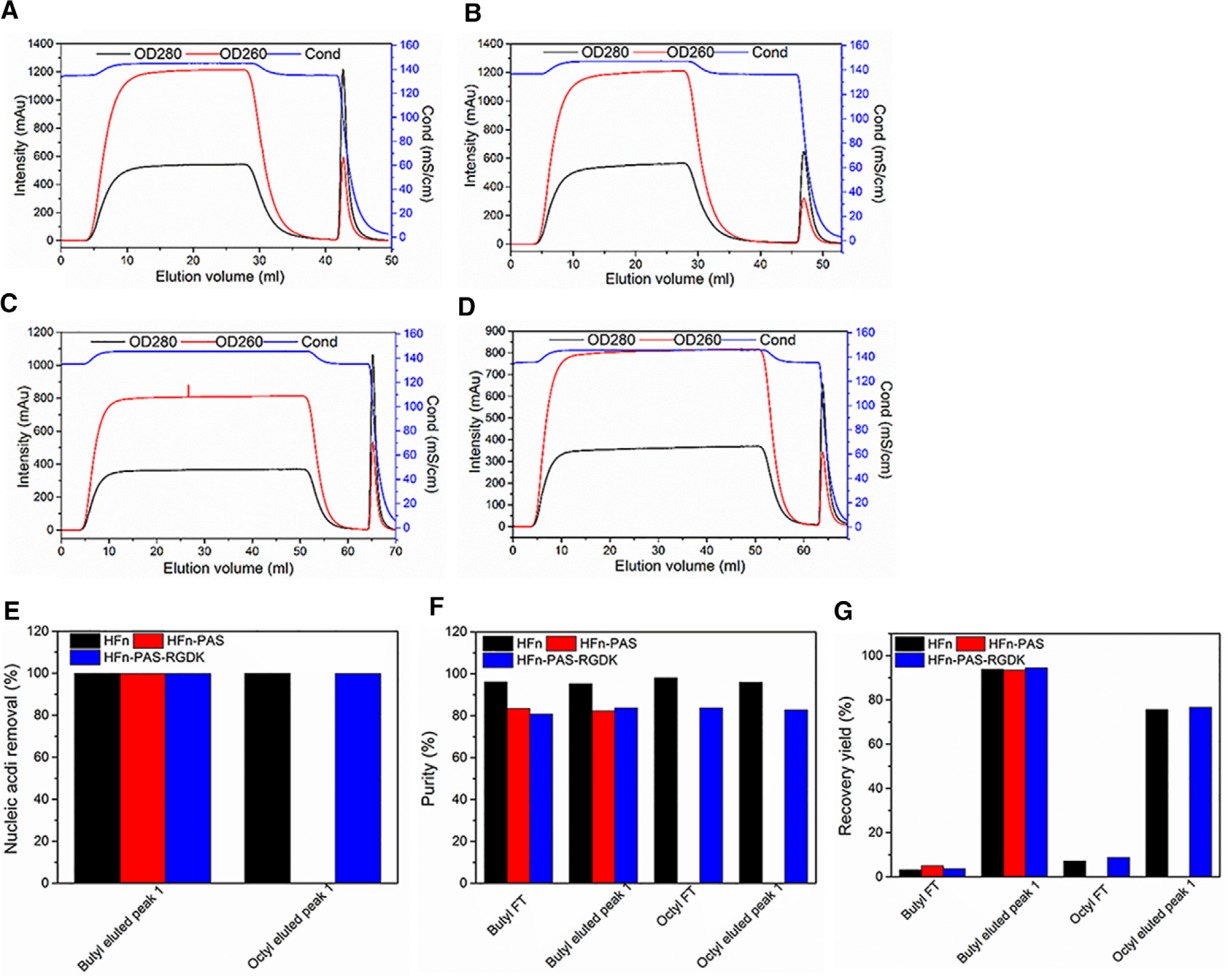
HIC chromatography purification of HFn and modified HFns. (A) HFn butyl FF chromatogram. (B) HFn octyl FF chromatogram. (C) HFn‐PAS‐RGDK butyl FF chromatogram. (D) HFn‐PAS‐RGDK octyl FF chromatogram. (E) Nucleic acid removal in HIC eluted peaks. (F) Purity in HIC peaks. (G) Recovery yield (step) in HIC peaks

In HIC, the strategy is the binding of target proteins and the flow through of nucleic acids. Nucleic acids are highly hydrophilic and hypothetically cannot bind to hydrophobic interaction columns. HFn, by contrast, has been proven to be able to bind to hydrophobic interaction column in a previous study [28]. Figure 4 includes the chromatograms of HIC, and the purity, recovery yield (step) and nucleic removal. Figure S4 is the SDS‐PAGE images of peaks from HIC and Table S3 lists the detailed purity, recovery yield (step) and nucleic acid removal data. As shown in Figure 4A‐D, OD260 nm of the FT peaks from butyl and octyl columns were higher than twice of OD280, suggesting they were mainly composed of nucleic acids. The eluted peaks containing target proteins, on the contrary, had higher OD280 than OD260. Both butyl and octyl FF achieved an above 98% nucleic acid removal for HFn and HFn‐PAS‐RGDK (Figure 4E), which was significantly higher than in Q FF IEC. This indicates HIC is very efficient in nucleic acid removal. Purity after HIC was above 95% for HFn and only above 82% for modified HFns (Figure 4F).

The recovery yield (step) of eluted peak in butyl FF HIC was above 90%, in comparison with above 80% in octyl FF HIC (Figure 4G). Because of the higher protein recovery yield (step), butyl FF HIC was selected for the second step of purification and employed for HFn‐PAS. As is presented in Figure 4G, both modified HFns had similar recovery yields (step) to that of HFn in butyl FF HIC, showing that the inserted PAS and RGDK peptide did not make a difference. The reasons are very likely to be: 1) both the inserted functional peptides were at the C‐terminal of the E‐helix, which does not affect the 4‐helix bundle residues constituting HFn nanocage outer surface, and 2) both functional peptides were random coils and hydrophilic which do not interact with resins.

Consequently, the final purification pathway consists of heat‐acid precipitation and butyl FF chromatography. A condition difference in heat‐acid precipitation existed in modified HFns in contrast of HFn because of the decline of stability against heat and acidic pH after fusion. Recovery yields (overall) after two‐step purification were: 92.98% (HFn), 63.93% (HFn‐PAS), and 58.09% (HFn‐PAS‐RGDK), respectively. The final protein amounts per gram of wet *E. coli* cell were 32.25 mg of HFn, 20.72 mg of HFn‐PAS and 20.22 mg of HFn‐PAS‐RGDK. Final purities were above 82% for modified HFns and above 95% for HFn. Modified HFns had lower purity and recovery yield (overall) primarily due to the stability decrease demonstrated in heat‐acid precipitation step. Nucleic acid removal by butyl FF HIC decreased nucleic acid amounts from 2000–3000 µg in loading sample to 2–4 µg in the final products.

### 3.4 Impact of modifications on HFn conformation

**FIGURE 5:**
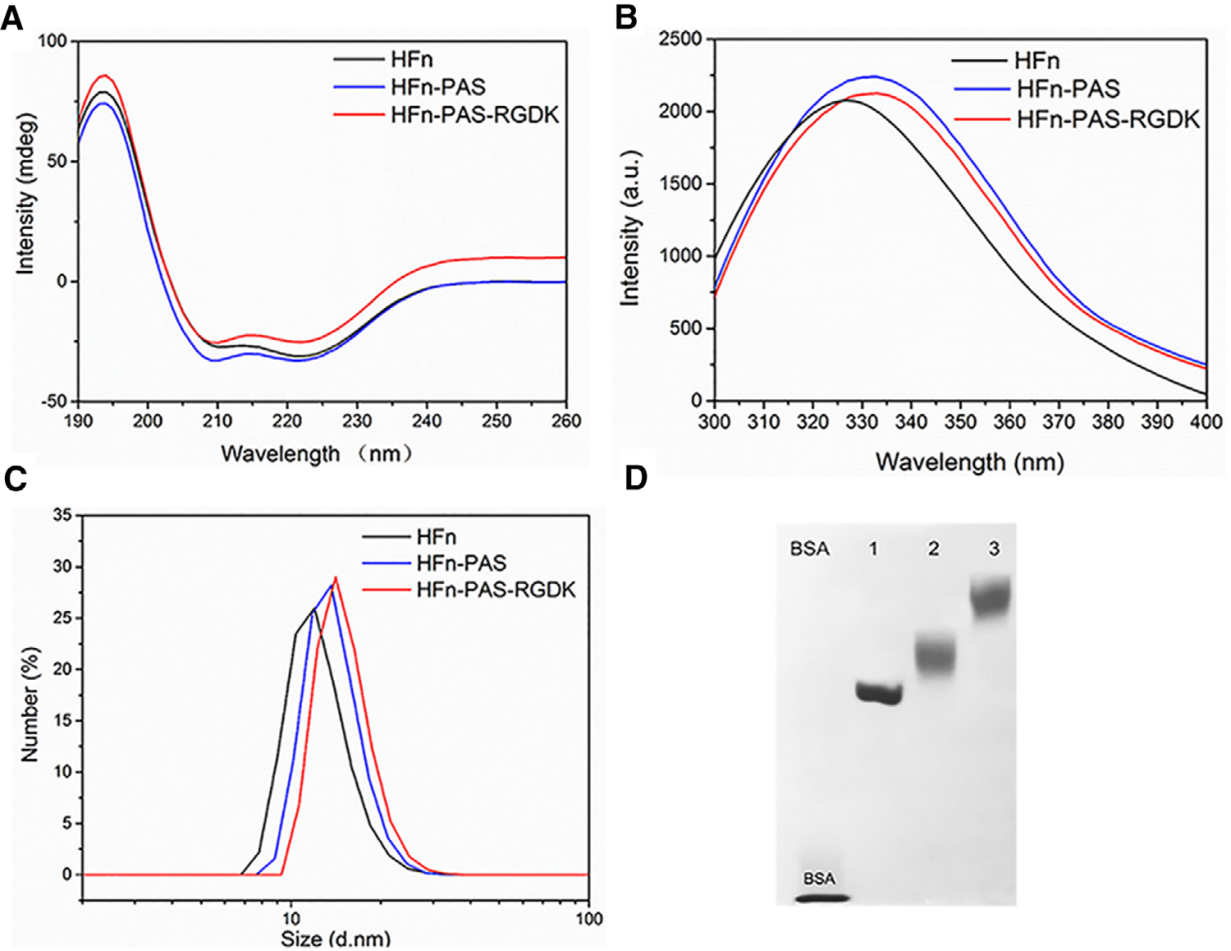
Structural characterization comparisons of HFn and modified HFns. (A) CD spectra. (B) IF spectra. (C) DLS hydrodynamic size by number. (D) 5% Native‐PAGE. Lane 1: HFn. 2, HFn‐PAS, 3: HFn‐PAS‐RGDK

The conformational differences between HFn and genetically modified HFns were investigated at secondary, tertiary and nanoparticle level to reveal the impact of modification and possible mechanism behind purification performance. Far‐UV CD analysis aimed to compare the secondary structure of modified HFns with that of HFn. In HFn subunit, approximate 68% residues form α‐helix and the rest are non‐structured loops [27]. As shown in Figure 5A, similar CD profiles indicate the protein conformation did not undergo significant secondary structural changes after fusion. Two negative bands at 208 and 222 nm prove that all three proteins are mainly α‐helix. This is because flexible linker and functional peptides are random coils, and fused to one end of HFn subunit, without disrupting subunit main structure of 4‐helix bundle conformation.

However, in the IF spectra (Figure 5B), both modified HFns (peaked at 333 nm) have a 7 nm red shift compared to HFn (326 nm). Protein IF spectrum is related to the microenvironment of Trp, Try, and Phe residues [29]. This red shift is probably caused by the polarity increase of microenvironment, which results from a conformational change in proteins [30]. This indicates that modified HFns have a conformational difference from HFn due to the C‐terminal insertion.

Protein hydrodynamic sizes and zeta potentials were measured by DLS (Figure 5C). The hydrodynamic sizes of HFn, HFn‐PAS and HFn‐PAS‐RGDK are 11.9, 13.7, and 14.1 nm, which proves that 24 subunits of all three proteins remained as self‐assemblies after purification. Inserted PAS and RGDK both increase protein hydrodynamic size, and PAS leads to a larger size increase than RGDK. Surface charges of proteins detected in zeta potential arise primarily from ionization of surface groups, such as acidic and basic side chains of exposed amino acid residues and associated counter ions bounding to protein surface [31]. At pH 7, a minor zeta‐potential difference between HFn (‐7.98 ± 0.23) and the modified HFns (HFn‐PAS: ‐5.73 ± 0.40, HFn‐PAS‐RGDK: ‐5.21 ± 0.22) was detected.

In Native‐PAGE analysis, proteins remain in natural form and the migration distances depend on both hydrodynamic size and surface charge density. HFn migrated the fastest and HFn‐PAS ranked the second (Figure 5D), followed by HFn‐PAS‐RGDK. It agrees with the hydrodynamic size order detected in DLS. Both modified HFns show relatively fuzzy bands than HFn, which possibly be ascribed to the non‐structured PAS peptides and linkers. The increase of hydrodynamic size caused by PAS can theoretically extend the half‐life of HFn in blood circulation, as was proven in previous studies [16, 17].

**FIGURE 6:**
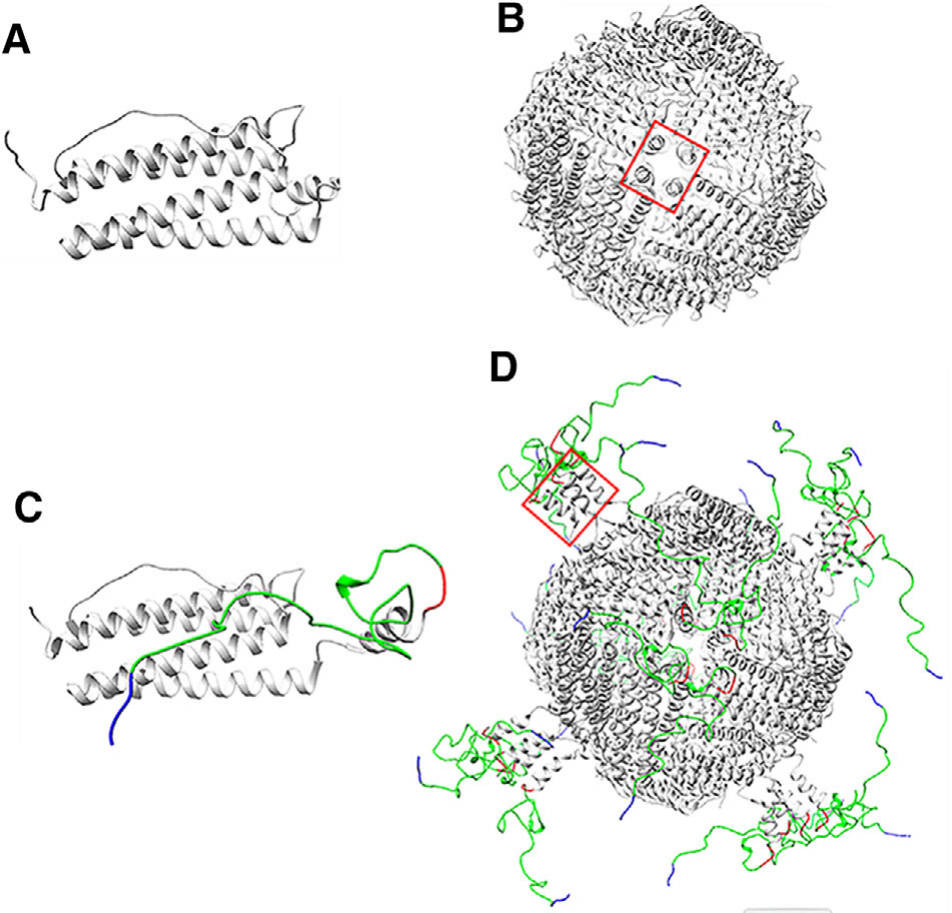
Schematic illustration of assumed protein structures. (A) “flip” HFn subunit. (B) “flip” HFn assembly. (C) “flop” modified subunit (HFn‐PAS‐RGDK). (D) “flop” assembly. Light grey parts represent HFn, red parts represent enzyme‐cleavable sequence GFLG, green parts are PAS peptides and blue parts are RGDK peptides. Structure of HFn was originally obtained from PDB (5N27) and then modified and visualized using Chimera. [27] Red rectangle indicates E‐helix location

Combing the results in purification and structural characterizations, there emerged two interesting findings. 1) The 12 nm of modified HFns and HFn spheres observed under TEM contradicts the different hydrodynamic sizes detected in DLS. 2) Binding strengths of modified HFns to Q FF column were generally higher than HFn although their predicted PI is higher than HFn PI, as the recovery yields (step) of FT peaks in Table S2. We therefore, reasonably speculate that some or all of the inserted peptides together with the E‐helix are exposed outside HFn nanocage. In this case, the exposed PAS peptides can bind to surrounding water molecules, so the hydrodynamic size of modified HFns were enlarged whilst the nanocage diameters observed under TEM remained the same as HFn. Binding strength to Q FF column has to do with the local negative charge density of the potential binding sites. The exposed E‐helices in modified HFns are probably responsible for the increase of the binding strength to Q FF column, because predicted PI of E‐helix is 3.84. Predicted PI values of E‐helix with PAS and E‐helix with PAS‐RGDK are 3.84 and 4.2, respectively. All these PI values are lower than HFn PI. This turnover of E‐helix has been discovered in previous studies and called the “flip‐flop” turnover [32]. The native HFn conformation with 24 E‐helices buried inside is called “flip” conformation (Figure 6A,B). If all 24 E‐helices are extruded outside nanocage, it is called “flop” conformation. Theoretical structures of subunits and assemblies in “flop” conformation are as shown in Figure 6C,D. The “flip‐flop” turnover happens when ferritin C‐terminal is inserted with large foreign peptide that cannot be accommodated by cavity. HFn with a red fluorescence protein (25.9 kDa) or 36 aa intrinsically disordered protein at C‐terminal have been proven to be in “flop” conformation [33, 34].

Average volume of residues is about 100 Å^3^ and average residue molecular weight is about 100 Da. The inner cavity of ferritin is 8 nm across and thus it can only accommodate 24 foreign peptides (maximum Mw = 9.2 kDa) when peptide structure and repulsive forces between peptides are not considered. Although the inserted PAS‐RGDK or PAS weights 5–6 kDa, the water‐binding ability of PAS peptides can make it impossible for HFn cavity to accommodate 24 inserted peptides simultaneously.

This putative turnover of the E‐helix with the inserted functional peptide could be the cause for the structural difference detected in tertiary structure and the decrease of stability discovered in heat‐acid precipitation. This is because, in “flip” conformation, the hydrophobic interactions between 4 E‐helix surrounding the hydrophobic channels have been proven to contribute to the stability of HFn assembly [32]. In the “flop” conformation; however, the 4 E‐helix are not physically confined as a bundle, but more likely to be floating around, leading to reduced hydrophobic interactions. Additionally, the spatial hindrance of the inserted water‐binding PAS peptides can further reduce the hydrophobic attractions between E‐helices.

### 3.5 In vitro performance comparison

**FIGURE 7:**
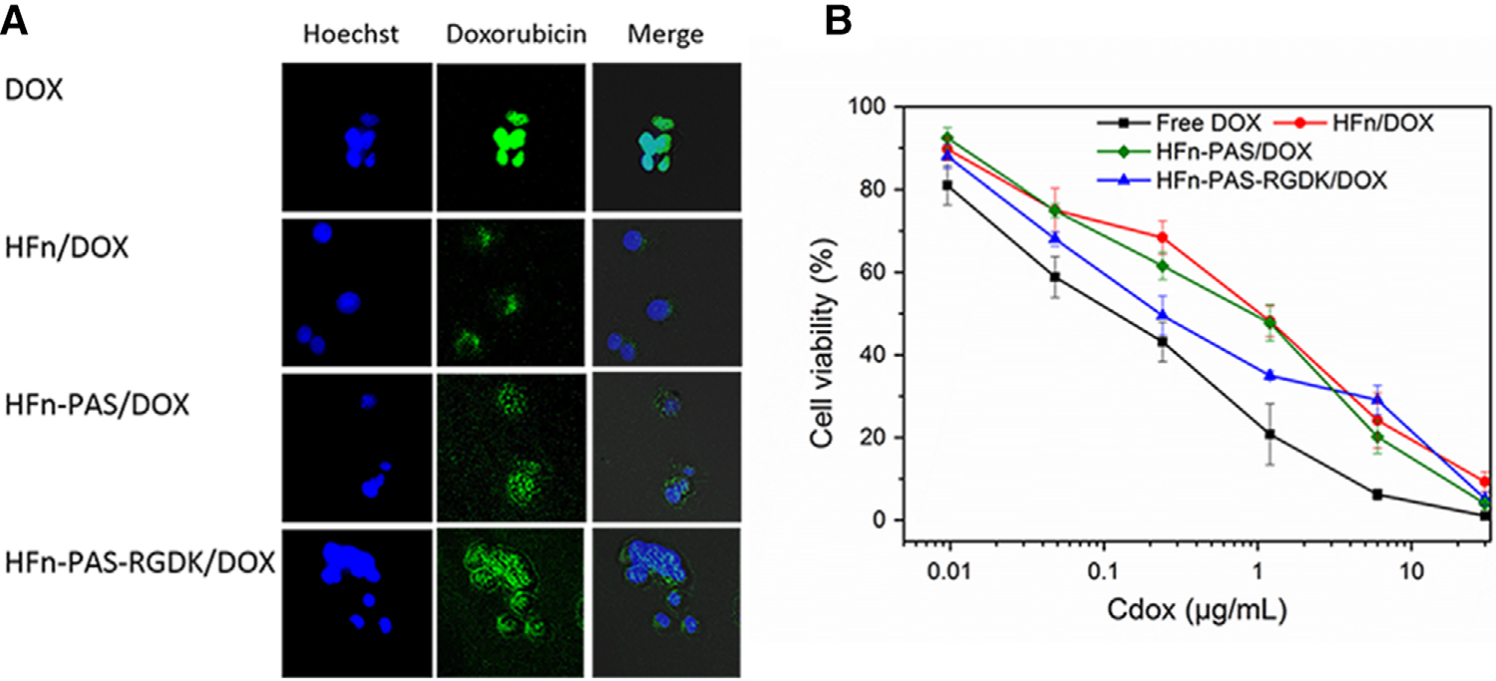
In vitro assessments of DOX loaded HFn and modified HFns. (A) Intracellular distribution images of all groups. Hoechst channel demonstrates the location of tumor cell nucleus by Hoechst 33258 staining of cell nucleus, and doxorubicin channel shows DOX distribution in tumor cells. (B) Anti‐proliferation effects of all groups on MCF7 cells. Data are represented as mean ± standard deviation, n = 3

After DOX loading, on average, 40.7, 39.3, 42.4 DOX molecules were loaded on HFn, HFn‐PAS and HFn‐PAS‐RGDK, respectively. Figure 7A shows the DOX distribution after entering MCF7 cells. In all groups, DOX accumulated in cell nucleus, but HFn and modified HFn groups showed a relatively low intensity in contrast with in DOX group. This could be due to the different cellular uptake mechanisms in different groups. Passive diffusion of DOX has been found to have a higher internalization efficiency in in vitro tests, compared with the receptor‐mediated cellular internalization in HFn‐based protein groups [18].

Figure 7B and Table S4 demonstrate the cytotoxicity results. DOX IC_50_ (0.20 ± 0.03 µg/mL) was significantly lower than all the other groups (*P* < 0.05). This could also be ascribed to the efficiency difference of cellular uptake approaches. IC_50_ of DOX/HFn‐PAS‐RGDK (0.59 ± 0.05 µg/mL) was significant lower than DOX/HFn (1.31 ± 0.22 µg/mL) and DOX/HFn‐PAS (0.98 ± 0.14 µg/mL), indicating RGDK peptide has enhanced the cytotoxicity through its affinity to tumor overexpressed integrin αvβ3 and neuropilin‐1 receptors [35]. This finding supports the possibility of the putative turnover of E‐helix, because the internalization requires the exposure of RGDK peptide. DOX/HFn‐PAS and DOX/HFn cytotoxicity were the lowest and their IC_50_ were not statistically different (*P* = 0.16), showing that PAS insertion has a negligible influence.

## 4 CONCLUDING REMARKS

HFn and two modified HFns, HFn‐PAS and HFn‐PAS‐RGDK were expressed as self‐assembled nanoparticles with high levels in *E. coli*. An effective two‐step purification pathway, heat‐acid precipitation plus butyl FF HIC, has been established for HFn. Heat‐acid precipitation removed HCPs and butyl FF HIC efficiently separated nucleic acids. However, a significantly lower stability of modified HFns has caused a final purity and recovery yield (overall) decline when adopting HFn purification process in modified HFns purification. Structural characterization shows that the stability decrease probably comes from the E‐helix turnover after PAS peptide insertion. PAS peptide has also resulted in an enlargement of hydrodynamic radius because of its hydration property. RGDK functionalization has shown a negligible impact on purification behavior by comparing HFn‐PAS and HFn‐PAS‐RGDK. In vitro tests; however, show that RGDK peptide has enhanced drug loaded HFn cytotoxicity.

## CONFLICT OF INTEREST

The authors have declared no conflict of interest.

## NOMENCLATURE

**Table:** 

Symbol	Explanation
DOX	Doxorubicin hydrochloride
DOX/HFn	HFn loaded with DOX
DOX/HFn‐PAS	HFn‐PAS loaded with DOX
DOX/HFn‐PAS‐RGDK	HFn‐PAS‐RGDK loaded with DOX

## Supporting information

Supporting information.

## Data Availability

The data that support the findings of this study are available from the corresponding author upon reasonable request.
