# Supplementary material for: Development of purification process for dual‐function recombinant human heavy‐chain ferritin by the investigation of genetic modification impact on conformation
Source: Eng Life Sci. 2021 Jun 19;21(10):630–42. doi: 10.1002/elsc.202000105 (PMC8518560; doi:10.1002/elsc.202000105)
Supplement: Supplementary file 1 — Supporting information. [file ELSC-21-630-s001.pdf]

Supporting information

Development of purification process for dual-function recombinant human heavy-chain ferritin by the investigation of genetic modification impact on conformation

Shuang Yin[1],

Bingyang Zhang[1],

Jianying Lin[2]

Yongdong Liu[3],

Zhiguo Su[3],

Jingxiu Bi[1].

1. School of Chemical Engineering & Advanced Materials, Faculty of Engineering, Computer and Mathematical Sciences, University of Adelaide, Adelaide, Australia

2. College of Biomedical Engineering, Taiyuan University of Technology

3. State Key Laboratory of Biochemistry Engineering, Institute of Process Engineering, Chinese Academy of Sciences, Beijing, China

Correspondence: A/Prof Jingxiu Bi (jingxiu.bi@adelaide.edu.au). School of Chemical Engineering &Advanced Materials, Faculty of Engineering, Computer and Mathematical Sciences, University of Adelaide, North terrace, SA5005, Adelaide, Australia.

Figure S1. 12 % reducing SDS-PAGE images of expression results of HFn and two modified HFns.

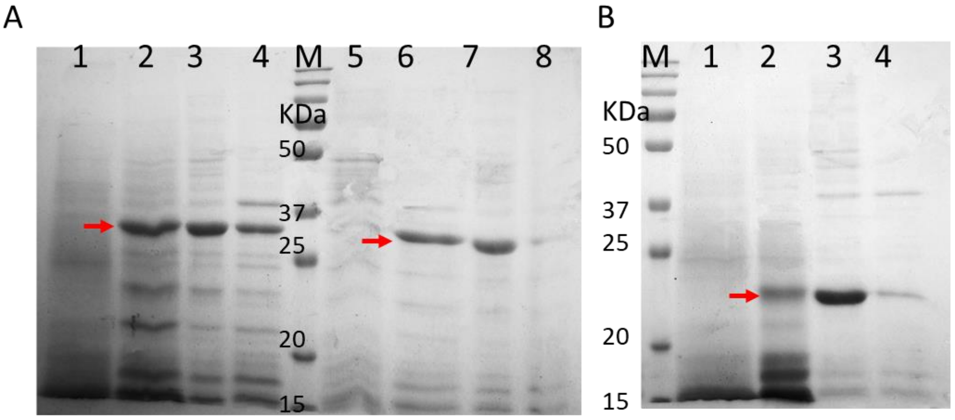

A, expression results of HFn-PAS and HFn-PAS-RGDK, lane 1: bacterial for HFn-PAS-RGDK before IPTG induction; lane 2: bacterial for HFn-PAS-RGDK after IPTG induction; lane 3: supernatant of bacterial lysate; lane 4: sediment of bacterial lysate; M:protein marker, lane 5: bacterial for HFn-PAS before IPTG induction; lane 6: bacterial for HFn-PAS after IPTG induction; lane 7: supernatant of bacterial lysate; lane 8: sediment of bacterial lysate. M: protein marker. **B**, expression result of HFn, lane 1: bacterial before IPTG induction; lane 2: bacterial after IPTG induction; lane 3: supernatant of bacterial lysate; lane 4: sediment of bacterial lysate. M: protein marker. Red arrows indicate target protein bands.

Figure S2. Q FF chromatograms of HFn and modified HFns.

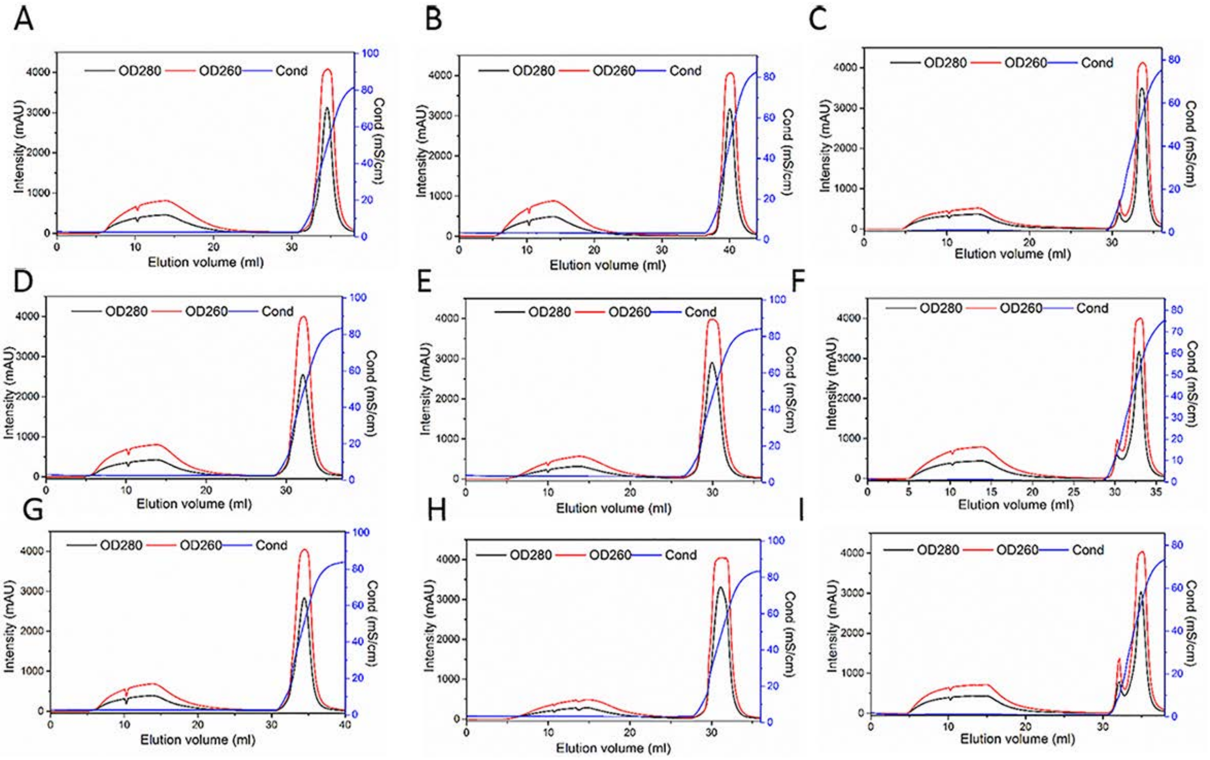

A, pH 7 HFn chromatogram. **B**, pH 8 HFn chromatogram. **C**, pH 9 HFn chromatogram. **D**, pH 7 HFn-PAS chromatogram. **E**, pH 8 HFn-PAS chromatogram. **F**, pH 9 HFn-PAS chromatogram **G**, pH 7 HFn-PAS-RGDK chromatogram. **H**, pH 8 HFn-PAS-RGDK chromatogram. **I**, pH 9 HFn-PAS-RGDK chromatogram.

Figure S3. 12 % reducing SDS-PAGE images of Q FF chromatography

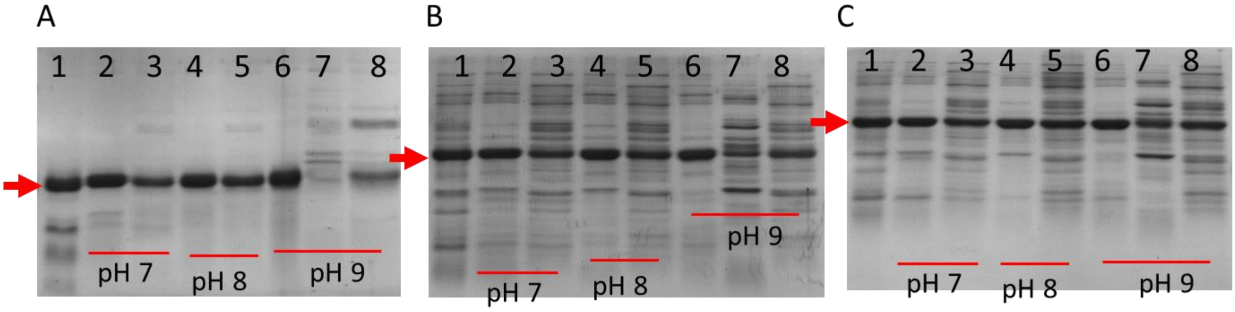

A, 12 % reducing SDS-PAGE results of HFn by Q FF chromatography. **B**, 12 % reducing SDS-PAGE results of HFn-PAS by Q FF chromatography. **C**, 12 % reducing SDS-PAGE results of HFn-PAS-RGDK by Q FF chromatography. Lane 1: heat-acid precipitation supernatant; lane 2: pH 7 flow through peak; lane 3: pH 7 eluted peak 1; lane 4: pH 8 flow through peak; lane 5: pH 8 eluted peak 1; lane 6: pH 9 flow through peak; lane 7: pH 9 eluted peak 1; lane 8: pH 9 eluted peak 2. Red arrows indicate target protein bands.

Figure S4. 12 % reducing SDS-PAGE images of HIC

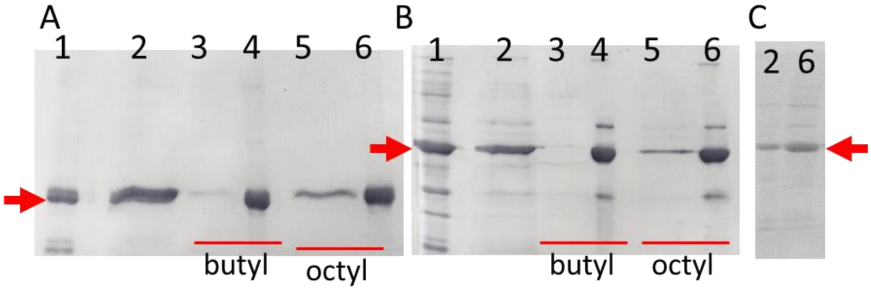

A, 12 % reducing SDS-PAGE results of peaks from HFn HIC. **B**, 12 % reducing SDS-PAGE results of peaks from HFn-PAS-RGDK HIC. **C**, 12 % reducing SDS-PAGE results of peaks from HFn-PAS butyl FF HIC. Lane 1: heat-acid precipitation supernatant; lane 2: Loading sample for HIC; lane 3: flow through peak of butyl FF; lane 4: eluted peak of butyl FF; lane 5: flow through peak of octyl FF; lane 6: eluted peak of octyl FF. Red arrows indicate target protein bands.

Table S1. Purity and recovery yield (step) in heat-acid precipitation

Protein	Conditions	50 ºC pH 4.0	50 ºC pH 4.5	50 ºC pH 5.0	60 ºC pH 4.5	60 ºC pH 5.0
HFn	Purity (%)	**64.77**	55.77	47.87	**65.50**	58.92
	Recovery yield (%)	88.70	91.58	93.82	**99.69**	99.22
HFn-PAS	Purity (%)	27.31	41.43	**50.77**	15.02	45. 01
	Recovery yield (%)	4.98	13.14	**68.41**	2.26	8.56
HFn-PAS-	Purity (%)	40.85	43.98	**50.41**	9.50	41.10
RGDK	Recovery yield (%)	0.32	12.55	**61.45**	0.88	9.19

Recovery yield (step) calculation example:

After 5 min heating at 60 ºC pH 4.5, HFn recovery yield (step) was:

Recovery yield (step) (%) = the amount HFn in heat-acid supernatant/ the amount of HFn in sample before heat-acid precipitation = (purity of HFn in heat-acid supernatant × total protein amount of heat-acid supernatant)/(purity of HFn in bacterial lysate (supernatant) × total protein amount of sample before heat-acid precipitation = (65.503 % × 0.5 mL × 2.058 mg/mL)/(33.806 % × 0.5 mL × 4.000 mg/mL) = 99.69 %

Table S2. Purity, recovery yield (step) and nucleic acid removal of peaks in Q FF IEC

**Protein**	**Q FF Peak**	**pH 7 FT**	**pH 7 P1**	**pH 8 FT**	**pH 8 P1**	**pH 9 FT**	**pH 9 P1**	**pH 9 P2**
HFn	Purity (%)	94.27	85.69	95.56	95.08	**97.98**	18.00	67.73
	Recovery yield (%)	82.12	16.34	80. 07	18.47	**91.68**	0.14	2.01
	Nucleic acid removal (%)	/	/	/	/	**46.00**	/	/
HFn-	Purity (%)	70.96	50.45	70.95	44.95	**80.13**	16.41	42.06
PAS	Recovery yield (%)	81.40	17.17	68.93	21.33	**79.06**	1.59	13. 83
	Nucleic acid removal (%)	/	/	/	/	**65.17**	/	/
HFn-	Purity (%)	69.92	51.72	75.60	49.40	**83.96**	32.75	45.96
PAS-	Recovery yield (%)	63.47	23.17	69.17	22.21	**70.25**	2.67	13.55
RGDK	Nucleic acid removal (%)	/	/	/	/	**46.81**	/	/

10 mg protein loaded in each run. FT: flow through peak. /: no measurement

Table S3. Purity, recovery yield (step) and nucleic acid removal of peaks in HIC

**Protein**	**Chromatography peak**	**Butyl FF FT**	**Butyl FF P1**	**Octyl FF FT**	**Octyl FF P1**
HFn	Purity (%)	96.09	**95.24**	98.02	95.94
	Recovery yield (%)	3.18	**93.87**	7.13	75.65
	Nucleic acid removal (%)	/	**99.93**	/	99.95
HFn-PAS	Purity (%)	83.48	**82.32**	/	/
	Recovery yield (%)	5.05	**93.46**	/	/
	Nucleic acid removal (%)	/	**99.81**	/	/
HFn-PAS-RGDK	Purity (%)	80.78	**83.74**	83.75	82.83
	Recovery yield (%)	3.68	**94.54**	8.79	76.66
	Nucleic acid removal (%)	/	**99.81**	/	99.78

10 mg protein loaded in each run. FT: flow through peak. /: no measurement.
Purity of FT peak is not as highly accurate as eluted peak purity because of the much lighter bands in gel.

Table S4. IC_{50} values of all groups against MCF7

**Group**	**IC_{50} (μg/mL)**
Doxorubicin	0.20±0.03
DOX/HFn	1.31±0.22
DOX/HFn-PAS	0.98±0.14
DOX/HFn-PAS-RGDK	0.59±0.05
